# Acute myocardial infarction in a patient with HAM/TSP evaluated for circulatory dynamics during mobilization before and after antihypertensive drug modification: a case report

**DOI:** 10.1186/s13256-026-06045-4

**Published:** 2026-05-29

**Authors:** Manami Sakai, Kenichi Fudeyasu, Toru Ueda, Yosuke Nakazato, Yoshinobu Matsumoto, Toshikazu Ohigashi, Kiyomichi Yoshimaru, Fumiko Yamaguchi

**Affiliations:** 1 Department of Rehabilitation, Osaka Saiseikai Ibaraki Hospital, 2-1-45, Mitsukeyama, Ibaraki, Osaka 567-0035 Japan; 2 Department of Rehabilitation, Osaka Medical and Pharmaceutical University Hospital, 2-7, Daigaku-Machi, Takatsuki, Osaka 569-8686 Japan; 3 Department of Pharmacy, Osaka Saiseikai Ibaraki Hospital, 2-1-45, Mitsukeyama, Ibaraki, Osaka 567-0035 Japan; 4 Department of Pharmaceutical Services, Hiroshima University Hospital, 1-2-3 Kasumi, Minami, Hiroshima 734-8551 Japan; 5 Department of Cardiology, Osaka Saiseikai Ibaraki Hospital, 2-1-45, Mitsukeyama, Ibaraki, Osaka 567-0035 Japan; 6 Department of Endocrinology and Diabetes, Osaka Saiseikai Ibaraki Hospital, 2-1-45, Mitsukeyama, Ibaraki, Osaka 567-0035 Japan

**Keywords:** Acute myocardial infarction, Orthostatic hypotension, Circulatory dynamics, Tilt table test, Antihypertensive therapy

## Abstract

**Background:**

Antihypertensive therapy plays a crucial role in managing cardiovascular disease but may sometimes cause significant hypotension during mobilization, hindering the recovery of activities of daily living. In patients struggling with orthostatic hypotension, evaluating circulatory dynamics using a tilt bed provides rich clinical insights for identifying the underlying pathophysiology. We encountered a rare case in which the patient also had HTLV-1-associated myelopathy/tropical spastic paraparesis (HAM/TSP), prompting an assessment of circulatory dynamics in the passive upright posture. We also aim to suggest that evaluating blood pressure responses using a tilt bed may provide valuable insights when antihypertensive management inadvertently limits functional improvement.

**Case presentation:**

A 74-year-old Japanese man with diabetes, chronic kidney disease, and slowly progressive HAM/TSP was admitted with acute myocardial infarction. Despite successful percutaneous coronary intervention, early mobilization was repeatedly interrupted by significant drops in blood pressure, leading to episodes of decreased consciousness. While resting blood pressure remained stable, passive upright posture with a tilt bed revealed that before modifying his antihypertensive regimen, his systolic blood pressure initially dropped by approximately 15 mmHg immediately after tilting and continued to decline for up to 10 min, ultimately reaching a total decrease of approximately 20 mmHg from baseline. After switching from sacubitril/valsartan to enalapril, an initial drop of 10–15 mmHg was still observed in the passive upright posture; however, blood pressure soon began to recover, preventing sustained hypotension. Consequently, the patient no longer experienced pronounced hypotension upon standing, enabling a more intensive rehabilitation program that ultimately led to improved activities of daily living.

**Conclusions:**

Our findings highlight that evaluating circulatory dynamics using a tilt bed provides critical clinical insights for optimizing medication and rehabilitation in patients whose recovery is impeded by orthostatic hypotension. Furthermore, it is essential to recognize that circulatory dynamics during mobilization can change significantly following medication adjustments. This approach may facilitate early mobilization and help mitigate prolonged hypotension that limits functional gains in selected patients.

**Supplementary Information:**

The online version contains supplementary material available at 10.1186/s13256-026-06045-4.

## Background

Early mobilization is crucial for improving outcomes in critically ill patients, including those with acute myocardial infarction (AMI) [1–3]. Orthostatic tolerance is essential for early mobilization, and monitoring circulatory dynamics is necessary during its implementation. Tilt tables are often used to assess a patient's circulatory dynamics [4–6]. Upright posture with a tilt table is commonly performed to evaluate orthostatic hypotension in healthy individuals, the elderly, patients with neuromuscular diseases, and critically ill patients [4, 5, 7–9].

Meanwhile, with advances in pharmacology, optimal pharmacotherapy for post-AMI patients is continuously evolving, often requiring individualized interventions. Pharmacotherapy plays a key role in improving outcomes in these patients, and both the JCS 2018 Guideline on Diagnosis and Treatment of Acute Coronary Syndrome [10] and the 2023 ESC Guidelines for the Management of Acute Coronary Syndromes [11] recommend antithrombotic therapy, lipid-lowering therapy, beta-blockers, nitrates, calcium channel blockers, renin–angiotensin–aldosterone system inhibitors, and diabetes medications. Among these, beta-blockers, nitrates, calcium channel blockers, and renin–angiotensin–aldosterone system inhibitors can influence circulatory dynamics. Recently, angiotensin receptor–neprilysin inhibitors (ARNIs), which have a novel mechanism of action that simultaneously inhibits neprilysin (NEP) and the renin–angiotensin–aldosterone system (RAAS), have been increasingly prescribed [11, 12]. However, to the best of our knowledge, no reports have specifically examined the extent to which antihypertensive drugs prescribed for cardiovascular disease affect circulatory dynamics during upright posture, such as mobilization. In patients struggling with orthostatic hypotension, evaluating circulatory dynamics using a tilt bed provides rich clinical insights for identifying the underlying pathophysiology and optimizing pharmacological management.

We encountered a case of AMI complicated by HTLV-1-associated myelopathy/tropical spastic paraparesis (HAM/TSP), in which improvement in activities of daily living (ADL) was delayed due to prolonged blood pressure reduction during mobilization. Autonomic dysfunction due to HAM/TSP was suspected, and circulatory dynamics were evaluated using a tilt table during passive upright posture. Although the diagnostic criteria for orthostatic hypotension were not met at 3 min after passive upright posture, a sustained decrease in blood pressure was observed for 10 min. After switching antihypertensive medication, episodes of impaired consciousness during mobilization ceased, and the sustained decrease in blood pressure in the passive upright position also improved. With this medication adjustment, mobilization and intensive rehabilitation became feasible, ultimately leading to improved ADL. Here, we report this case of assessing circulatory dynamics in optimizing pharmacotherapy for mobilization.

## Case presentation

### Patient background

A 74-year-old Japanese man with a history of diabetes, hypertension, chronic kidney disease, and slowly progressive HAM/TSP was admitted due to loss of appetite, general malaise, and difficulty in movement. Before his illness, he lived alone, using a cane indoors and an electric wheelchair outdoors for 20 years due to myelopathy. His ex-wife, who lived next door, assisted him with shopping and meal preparation. He had a long-term care level of 2 and received home rehabilitation twice a week. His Barthel Index before illness was 85, with deductions for walking and stair climbing, and his Osame Motor Disability Score (OMDS), which assesses motor dysfunction severity in HAM/TSP on a scale of 0–13, was 7.

### Onset of myocardial infarction

On the day before admission, the patient was hospitalized for investigation and treatment. On the following day, he developed fever and impaired consciousness. A 12-lead electrocardiogram revealed ST elevation in leads II, III, and aVF, along with ST depression in leads aVL and V1–V2, leading to a diagnosis of AMI. Cardiogenic shock was present, but due to his general condition, transfer to a tertiary emergency hospital was deemed too risky. Percutaneous coronary intervention (PCI) and intra-aortic balloon pump (IABP) insertion were performed at our hospital, a secondary emergency and clinical training facility. The culprit lesion was #1, and PCI successfully improved the occlusion from 100% to 0% with no residual stenosis. Peak creatine kinase-MB was 195 U/L on day 0. The IABP was removed on day 2, and physical therapy was initiated on day 3 following consultation with the rehabilitation department.

### Pharmacotherapy

Post-AMI pharmacotherapy included the discontinuation of catecholamines on day 2, followed by the initiation of sacubitril/valsartan at 100 mg/day. Carvedilol at 5 mg/day was started on day 3, and spironolactone at 75 mg/day was added due to persistent heart failure symptoms. Spironolactone was discontinued on day 14 as heart failure symptoms improved. On day 19, paroxysmal supraventricular tachycardia at 120–130 bpm occurred every morning, prompting the initiation of verapamil hydrochloride at 40 mg/day and an increase in carvedilol to 10 mg/day.

In this regimen, persistent hypotension with impaired consciousness during mobilization led to a tilt test on day 46. On the following day, sacubitril/valsartan was discontinued and replaced with enalapril maleate at 1.25 mg/day. The decision to switch was based on the patient's prolonged hypotensive response observed during the tilt test, which could not be fully explained by his underlying HAM/TSP or other comorbidities. Sacubitril/valsartan, while generally beneficial in post-AMI patients, inhibits NEP, which enhances natriuretic peptide-mediated vasodilation. This mechanism was hypothesized to contribute to mobilization difficulties. Therefore, a trial switch to enalapril, an ACE inhibitor without NEP inhibition, was deemed necessary to assess its impact on circulatory dynamics during mobilization.

### Rehabilitation therapy

**Fig. 1:**
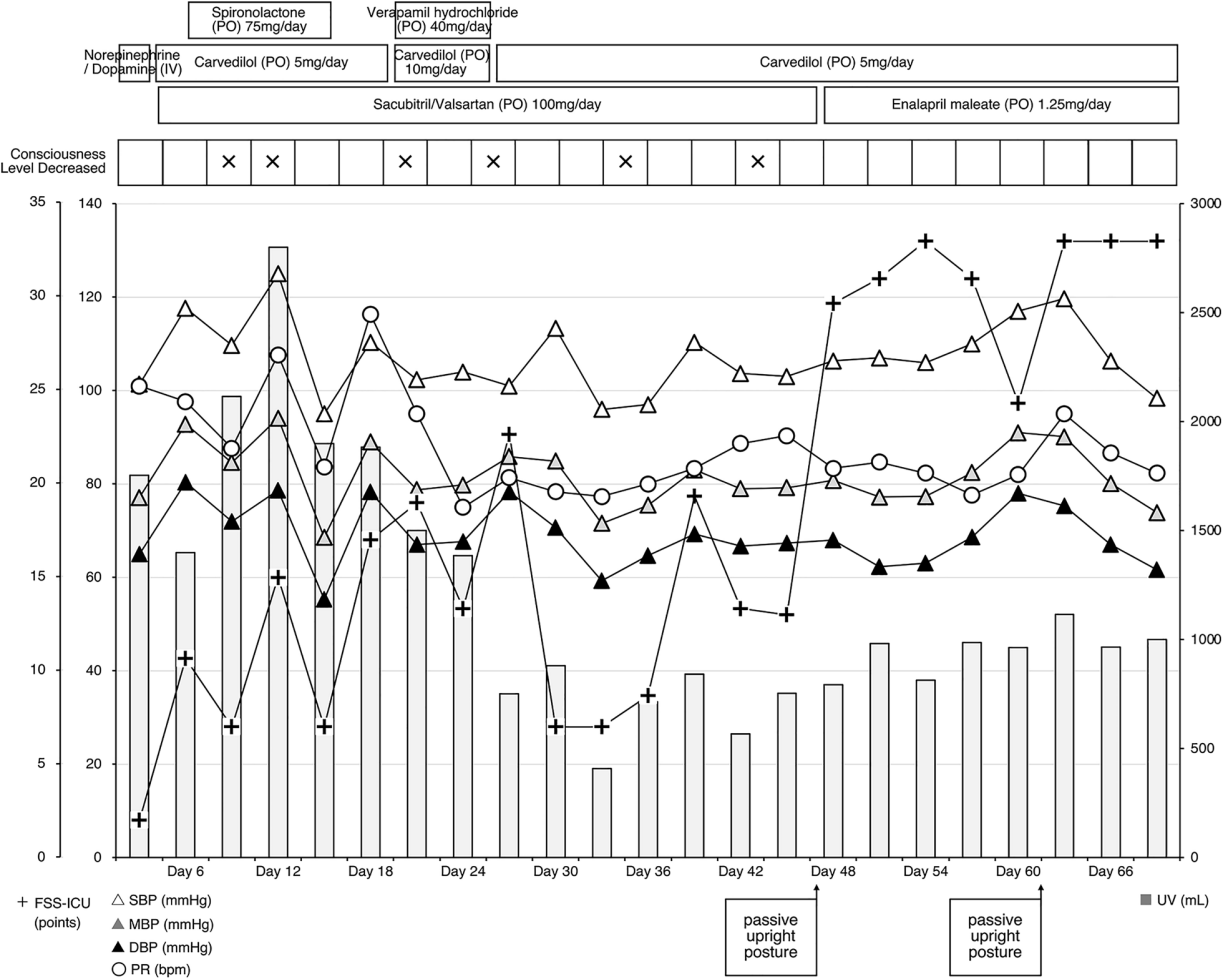
Changes in medication, vital signs, occurrence of decreased consciousness during physical therapy, and changes in ADL ability (FSS–ICU) from AMI onset through transfer. This figure illustrates the patient’s medication history after AMI, changes in vital signs and ADL ability every 3 days, and the presence or absence of decreased consciousness due to blood pressure drops during physical therapy. Sacubitril/valsartan was discontinued on day 46, and enalapril was administered from day 47. Before the medication change, frequent episodes of hypotension-related consciousness disturbances occurred during transfers (×). After switching to enalapril, these episodes ceased, and the FSS–ICU score, representing ADL function, began to improve

Rehabilitation followed a staged mobilization approach in accordance with the clinical pathway [13]. Although early ambulation is generally recommended for AMI patients, those with extensive myocardial damage, heart failure, hypotension, or arrhythmias may require extended bed rest or restricted physical activity [11]. Given the severity of this case, a cautious, stepwise rehabilitation strategy was implemented. However, frequent episodes of hypotension-induced impaired consciousness hindered intensive therapy, resulting in suboptimal ADL improvement. On day 46, the Barthel Index was 20, and the Functional Status Score for the ICU (FSS–ICU) was 13. Figure 1 illustrates the mean vital signs recorded every 3 days after PCI, the mean FSS–ICU as an indicator of ADL ability, and the presence or absence of impaired consciousness due to hypotension. Table 1 presents the trends in blood test results, while Table 2 shows the sequential changes in echocardiographic findings.

**Table 1:** Trends in blood data after AMI onset

	Day 0	Day 1	Day 2	Day 3	Day 5	Day 10	Day 20	Day 30	Day 40	Day 60
Lac (mmol/L)	5.9	2.0								
WBC (× 10^3^/µL)	154	222	154	124	146	203	77	79	75	64
HGB (g/dL)	13.8	12.3	11.7	11.9	12.2	12.5	11.4	11.4	10.5	9.9
PLT (× 10^3^/μL)	20.0	11.3	8.3	6.0	13.2	29.9	24.7	28.1	25.4	27.3
ALB (g/dL)	3.0		2.4			2.3	2.5			2.7
Cre (mg/dL)	1.11	1.65	1.06	0.79	0.79	0.79	0.68	0.91	0.80	0.72
CRP (mg/dL)	12.31		15.92			13.46	0.74	1.61	1.11	1.53
AST (U/L)	32	1840	892	272	69	29	39	20	16	11
ALT (U/L)	22	1049	887	509	236	82	56	18	11	8
T-Bil (mg/dL)	0.8	0.7	0.6	0.7	0.8	0.8	0.5	0.7	0.4	0.3
CK (U/L)	41	4898	1281	368		28	20			
CKMB (U/L)	195	173	67	36		4				
BNP (pg/mL)	75.9							180		158

*Lac* lactate, *WBC* white blood cell count, *HGB* hemoglobin, *PLT* platelet count, *ALB* albumin, *Cre* creatinine, *CRP* C-reactive protein, *AST* aspartate aminotransferase, *ALT* alanine aminotransferase, *T-Bil* total bilirubin, *CK* creatine kinase, *CK-MB*, creatine kinase-MB; *BNP* B-type natriuretic peptide

**Table 2:** Echocardiographic findings after AMI onset

	LVEF (%)	LVDd (mm)	LVDs (mm)	IVC exp–insp (mm)	TR	WMA	E/e’
Day 0	43	34	27	15 (respiratory variation −)	mild	inf.base–mid akinesis	
Day 5	55	42	30	12–4 (respiratory variation +)		inf.base–mid hypokinesis	8.0
Day 20	52	42	31	11–6 (respiratory variation +)	trivial	inf.base–apex akinesis	8.1
Day 40	50	49	36	12–6 (respiratory variation +)	mild	inf.base–mid akinesis	14.6
Day 60	54	48	35	10–4 (respiratory variation +)	mild	inf.base–mid severe hypo	11.2
Day 80	60	46	32	8–3 (respiratory variation +)	mild	inf.base–mid akinesis	6.2

*LVEF* left ventricular ejection fraction, *LVDd* left ventricular end-diastolic dimension, *LVDs* left ventricular internal dimension in systole, *IVC* inferior vena cava, *TR* tricuspid regurgitation, *WMA* wall motion abnormality

### Tilt table test and circulatory dynamics

Given the patient’s 20-year history of HAM/TSP, autonomic dysfunction was suspected. HAM/TSP is a chronic inflammatory spinal cord disease characterized primarily by spastic paraplegia due to HTLV-1 infection. It may also involve sensory, bladder, rectal, and autonomic dysfunction, including orthostatic hypotension [14, 15]. To evaluate circulatory dynamics, a tilt table test was performed on days 46 and 60 using a TILT TABLE UA-452 (OG Wellness Technologies Co., Ltd, Okayama, Japan).

Passive upright posture was maintained at 60° for 15 min following 15 min of supine rest, with subsequent evaluation of heart rate (HR), systolic blood pressure (SBP), diastolic blood pressure (DBP), mean blood pressure (MBP), respiratory rate (RR), and peripheral oxygen saturation (SpO2) at 1-min intervals, averaged every 3 min, using a bedside monitor PVM-4000 (Nihon Kohden Corporation, Tokyo, Japan). The patient did not report pain during the procedure.

**Fig. 2:**
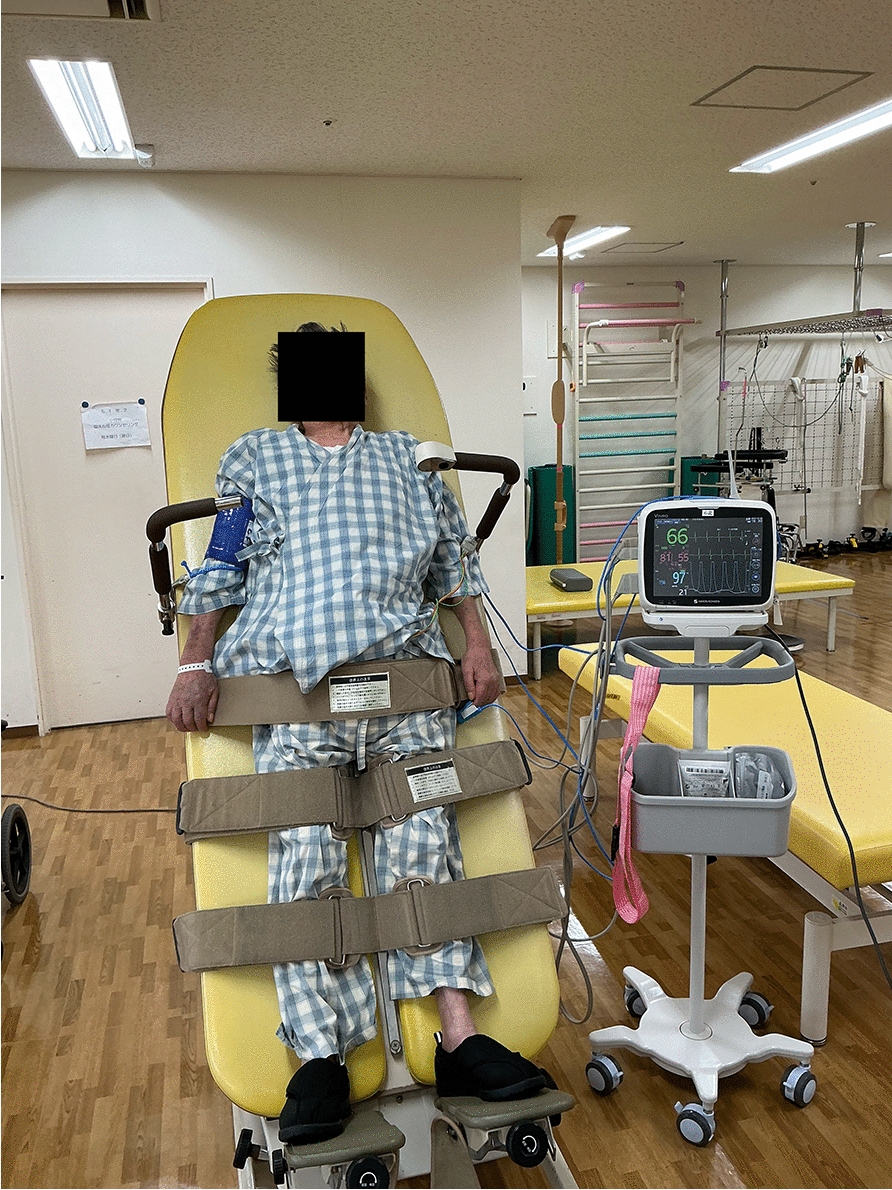
Circulatory dynamics on day 46 before changing the antihypertensive regimen. During passive upright posture, systolic blood pressure (SBP) dropped by approximately 15 mmHg immediately after tilting and continued to decline for nearly 10 min, eventually reaching a decrease of approximately 20 mmHg from baseline. Heart rate (HR) did not show a rapid compensatory increase

During the initial test, while on ARNI therapy, the mean SBP for the preceding 5 days was 105.8 mmHg. Upon assuming an upright posture, SBP decreased by approximately 15 mmHg within 3 min but did not meet the diagnostic criterion for orthostatic hypotension, which requires a drop of ≥ 20 mmHg [16]. However, SBP continued to decline over 10 min, and HR showed an unusually slow compensatory increase (Table 3; Fig. 2, Additional file 1). On day 47, sacubitril/valsartan was discontinued, and enalapril maleate was introduced. After this change, intensive rehabilitation was implemented without further hypotension-induced impaired consciousness during mobilization.

**Table 3:** Circulatory dynamics during passive upright posture before medication change (day 46)

	supine					upright posture					supine	
min	3	6	9	12	15	3	6	9	12	15	3	5
HR (bpm)	72.0	73.0	73.3	73.7	74.0	77.7	79.3	80.3	84.0	84.3	78.7	81.5
SBP (mmHg)	91.7	93.3	95.3	94.0	99.7	86.0	82.3	78.3	87.7	85.7	91.0	96.0
DBP (mmHg)	56.3	57.3	59.0	58.3	61.7	52.7	56.3	53.0	57.3	54.7	63.0	66.0
MBP (mmHg)	68.0	69.3	70.7	69.7	73.3	63.3	64.7	61.3	68.0	64.7	74.0	72.5
SpO2(%)	96.0	96.7	96.3	96.7	95.7	96.0	96.3	94.0	93.3	96.3	93.7	91.5
RR (bpm)	22.0	19.0	20.3	20.7	19.7	22.3	24.0	24.3	25.3	24.7	19.3	23.0

*HR* heart rate, *SBP* systolic blood pressure, *DBP* diastolic blood pressure, *MBP* mean blood pressure, *SpO*_*2*_ peripheral capillary oxygen saturation, *RR* respiratory rate

### Follow-up

**Fig. 3:**
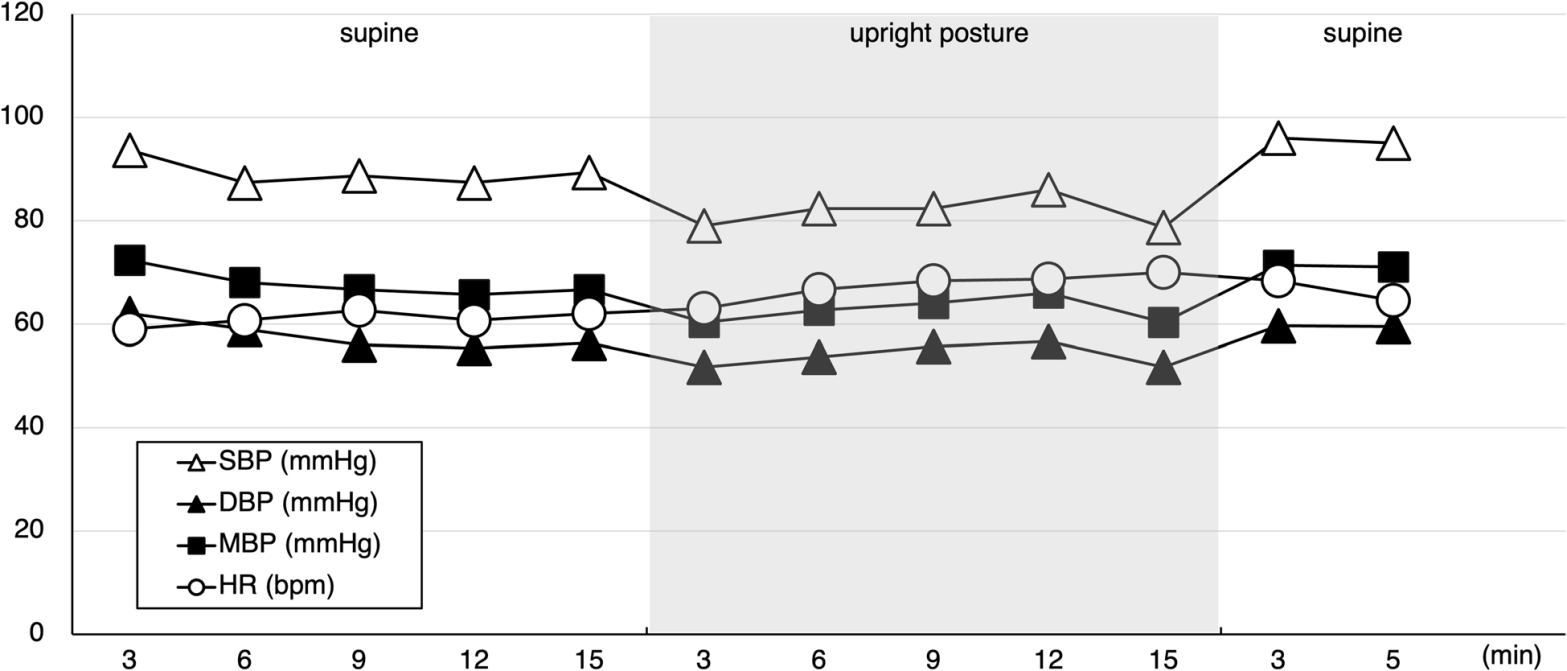
Circulatory dynamics on day 60 after changing the antihypertensive regimen. On day 60, the SBP initially decreased by about 10–15 mmHg upon tilting, similar to day 46. However, the SBP decline halted within a few minutes and began to rise slightly thereafter, resembling a more normal compensatory response. As in the earlier test, HR increase was gradual but did not contribute to ongoing hypotension

Following the medication adjustment, hypotensive episodes severe enough to impair consciousness no longer occurred. A repeat tilt table test on day 60 showed that SBP, which had previously continued to decrease for 10 min, now stabilized within 3 min and began to recover slightly. The mean SBP for the preceding 5 days was 113.4 mmHg, and the initial drop upon assuming an upright posture was limited to 10–15 mmHg, with a stabilization trend observed thereafter (Table 4; Fig. 3, Additional file 2). HR responses remained gradual, similar to pre-treatment levels.

**Table 4:** Circulatory dynamics during passive upright posture after medication change (day 60)

	Supine					Upright posture					Supine	
Min	3	6	9	12	15	3	6	9	12	15	3	5
HR (bpm)	59.0	60.7	62.7	60.7	62.0	63.0	66.7	68.3	68.7	70.0	68.3	64.5
SBP (mmHg)	93.7	87.3	88.7	87.3	89.3	79.0	82.3	82.3	86.0	78.7	96.0	95.0
DBP (mmHg)	62.0	59.0	56.0	55.3	56.3	51.7	53.7	55.7	56.7	51.7	59.7	59.5
MBP (mmHg)	72.3	68.0	66.7	65.7	66.7	60.3	62.7	64.0	66.0	60.3	71.3	71.0
SpO2(%)	97.7	95.7	94.3	95.0	95.7	98.0	96.7	94.3	96.0	95.7	94.0	95.5
RR (bpm)	19.7	19.7	21.7	20.7	18.7	23.0	23.7	23.3	22.7	22.0	19.7	20.5

*HR* heart rate, *SBP* systolic blood pressure, *DBP* diastolic blood pressure, *MBP* mean blood pressure, *SpO*_*2*_ peripheral capillary oxygen saturation, *RR* respiratory rate

ADL improved significantly, with the FSS–ICU increasing to 31 and the Barthel Index improving to 45 by day 60. The patient reported improved mobility and no further episodes of hypotension-related impaired consciousness following medication adjustment. On day 74, the patient was transferred to another hospital, and by day 141, ADL had improved sufficiently for hospital discharge.

## Conclusions and discussion

We encountered a case of AMI complicated by HAM/TSP, in which improvement in ADL was delayed due to a prolonged blood pressure drop during mobilization. After adjusting the patient's medication, mobilization and intensive rehabilitation became feasible, ultimately leading to improved ADL. To evaluate circulatory dynamics before and after the medication change, passive upright posture was assessed using a tilt table. Although a 5-min upright posture is recommended in the original tilt test [17], the present study maintained the upright posture for 15 min, which was deemed sufficient for interpreting the results [6].

HAM/TSP, a rare disease affecting 0.25–3.8% of HTLV-1 carriers, is characterized by spastic paraplegia, pathological reflexes, voiding dysfunction, mild paresthesia, and reduced sweating [18]. HTLV-1 is transmitted through breastfeeding, blood transfusion, and sexual contact. HAM/TSP can follow either a rapidly progressive course, leading to loss of ambulation within 2 years in approximately 30% of cases, or a slowly progressive course, which may take up to 15 years to cause ambulatory impairment [18]. It has been reported that orthostatic hypotension can occur in HAM/TSP even in the absence of subjective dizziness [14], possibly due to cardiac sympathetic nerve involvement [15]. In this case, the patient had the slowly progressive form of HAM/TSP, and autonomic dysfunction was suspected due to the prolonged hypotensive episodes. However, passive upright posture tests performed before and after the medication change revealed that SBP did not decrease by ≥ 20 mmHg, nor did DBP decrease by ≥ 10 mmHg within 3 min, indicating that the condition did not meet the criteria for orthostatic hypotension [16]. The rarity of this clinical presentation and the fact that tilt bed measurements were performed with a sufficient interval to minimize carryover effects suggest that this report could be viewed as an "n-of-1 trial" [19]. It provides a clinical subset of a crossover design, offering insights for individualized management.

Regarding circulatory responses to passive upright posture, studies in healthy individuals have shown that HR increases by approximately 10–15 bpm within a few minutes of standing [8], while SBP decreases by approximately 5–10 mmHg 1 min after standing but subsequently recovers [7]. In this case, SBP decreased by approximately 15 mmHg immediately after passive upright posture both before and after the medication change, a greater drop than typically observed in healthy individuals. Normally, α-adrenergic receptors mediate vasoconstriction, while β-adrenergic receptors increase HR and cardiac output during the transition from a supine to an upright posture. Since the patient had AMI, the use of carvedilol, a β-blocker with additional α-blocking effects, likely contributed to the insufficient compensatory response in HR and cardiac output, as well as inadequate peripheral vasoconstriction [20].

Following the medication change, SBP stopped decreasing within a few minutes of assuming an upright posture and showed a slight upward trend, a response more similar to that of healthy individuals. Before the medication change, however, SBP continued to decline for approximately 10 min. A reduction in renal blood flow and activation of β-sympathetic nerves typically stimulate the RAAS, leading to renin release. Angiotensin II, produced via RAAS activation, promotes vasoconstriction and sodium retention, stabilizing blood pressure during prolonged upright posture [21]. In patients with complete cervical spinal cord injury, postural hypotension has been reported to increase renin levels within minutes due to decreased renal blood flow [22].

Sacubitril/valsartan, an ARNI, has both RAAS-inhibitory and NEP-inhibitory effects. NEP degrades natriuretic peptides, which have vasodilatory properties, and its inhibition enhances vasodilation, resulting in a more pronounced antihypertensive effect compared to ACE inhibitors or angiotensin receptor blockers (ARBs) [12, 23]. In non-ST-elevation myocardial infarction (NSTEMI), ARNI has been shown to improve composite cardiovascular endpoints compared to ACE inhibitors [24]. However, ARNI has also been associated with a higher incidence of hypotension compared to ACE inhibitors in both NSTEMI and ST-elevation myocardial infarction (STEMI) [24]. Specifically, since sacubitril/valsartan 100 mg contains 51.4 mg of valsartan, we switched to enalapril 1.25 mg, which is expected to have a milder antihypertensive effect than 50 mg of valsartan. This switch aimed to maintain cardioprotective RAAS inhibition while removing the NEP inhibition hypothesized to cause excessive, sustained vasodilation. It is possible that the vasodilatory effect of ARNI counteracted the vasoconstriction that should have occurred continuously during passive upright posture.

In cases where resting blood pressure remains stable but mobilization is hindered, evaluating circulatory dynamics using a tilt bed provides rich clinical insights. Other potential causes of prolonged hypotension were considered, including autonomic failure unrelated to HAM/TSP, but the tilt test findings and pharmacological response supported our hypothesis. Importantly, our findings highlight that circulatory dynamics during mobilization can fluctuate following medication adjustments. This necessitates extended monitoring and reassessment to optimize treatment strategies for patients experiencing mobilization difficulties.

Some randomized controlled trials have excluded patients with orthostatic hypotension based on clinical history or objective measurements, which may have led to an underestimation of the risks of symptomatic hypotension, falls, and syncope in real-world clinical settings [25]. Orthostatic hypotension is not only an independent risk factor for cardiovascular morbidity and mortality [26, 27], but also a significant concern in rehabilitation, as it increases the risk of traumatic injuries [28]. A randomized controlled trial comparing community-dwelling older adults with orthostatic hypotension who either discontinued or continued antihypertensive therapy found that discontinuation was associated with resolution of orthostatic hypotension [29]. However, that study reported blood pressure changes upon transition from sitting to standing and did not employ a standardized protocol for diagnosing orthostatic hypotension. When faced with the dilemma of antihypertensive treatment versus orthostatic hypotension, particularly in cases where mobilization and ADL recovery are hindered, evaluating circulatory responses during passive upright posture using a tilt table, as in this case, may be beneficial.

There are several limitations to this case report. First, as a single case report, the generalizability of the findings is inherently limited. Second, SBP increased by 7.6 mmHg following the change in antihypertensive medication, making it difficult to rule out the possibility that this baseline increase contributed to improvements in physical therapy outcomes and the resolution of consciousness disturbances during mobilization.

Our findings highlight that evaluating circulatory dynamics using a tilt bed provides critical clinical insights for optimizing medication and rehabilitation in patients whose recovery is impeded by orthostatic hypotension. Furthermore, it is essential to recognize that circulatory dynamics during mobilization can change significantly following medication adjustments.

This case report was prepared in accordance with the CARE guidelines, and the completed CARE checklist is provided as Additional file 3.

## Supplementary Information

Additional file 1: Minute-by-minute circulatory dynamics during passive upright posture before medication change (Day 46)

Additional file 2: Minute-by-minute circulatory dynamics during passive upright posture after medication change (Day 60)

Additional file 3: CARE checklist

## Data Availability

The datasets used and/or analyzed during the current study are available from the corresponding author upon reasonable request.

## Abbreviations

ACE: Angiotensin-converting enzyme
ACS: Acute coronary syndrome
ADL: Activities of daily living
AMI: Acute myocardial infarction
ARB: Angiotensin receptor blocker
ARNI: Angiotensin receptor–neprilysin inhibitor
BP: Blood pressure
DBP: Diastolic blood pressure
eGFR: Estimated glomerular filtration rate
FSS-ICU: Functional Status Score for the Intensive Care Unit
HAM/TSP: HTLV-1-associated myelopathy/tropical spastic paraparesis
HR: Heart rate
HTLV-1: Human T-lymphotropic virus type 1
IABP: Intra-aortic balloon pump
MBP: Mean blood pressure
NEP: Neprilysin
NSTEMI: Non-ST-elevation myocardial infarction
OMDS: Osame Motor Disability Score
PCI: Percutaneous coronary intervention
RAAS: Renin–angiotensin–aldosterone system
RR: Respiratory rate
SBP: Systolic blood pressure
SpO₂: Peripheral oxygen saturation
STEMI: ST-elevation myocardial infarction
