# Supplementary material for: Acute myocardial infarction in a patient with HAM/TSP evaluated for circulatory dynamics during mobilization before and after antihypertensive drug modification: a case report
Source: J Med Case Rep. 2026 May 29;20:435. doi: 10.1186/s13256-026-06045-4 (PMC13422343; doi:10.1186/s13256-026-06045-4)
Supplement: Supplementary file 1 — Additional file 1: Minute-by-minute circulatory dynamics during passive upright posture before medication change (Day 46) [file 13256_2026_6045_MOESM1_ESM.docx]

**Supplemental 1. Minute-by-minute circulatory dynamics during passive upright posture before medication change (Day 46)**

|  | supine position | | | | | | | | | | | | | | | upright posture | | | | | | | | | | | | | | | supine position | | | | |
| --- | --- | --- | --- | --- | --- | --- | --- | --- | --- | --- | --- | --- | --- | --- | --- | --- | --- | --- | --- | --- | --- | --- | --- | --- | --- | --- | --- | --- | --- | --- | --- | --- | --- | --- | --- |
| min | 1 | 2 | 3 | 4 | 5 | 6 | 7 | 8 | 9 | 10 | 11 | 12 | 13 | 14 | 15 | 1 | 2 | 3 | 4 | 5 | 6 | 7 | 8 | 9 | 10 | 11 | 12 | 13 | 14 | 15 | 1 | 2 | 3 | 4 | 5 |
| HR (bpm) | 72 | 72 | 72 | 73 | 73 | 73 | 74 | 73 | 73 | 73 | 74 | 74 | 74 | 74 | 74 | 76 | 78 | 79 | 80 | 79 | 79 | 80 | 80 | 81 | 85 | 83 | 84 | 85 | 82 | 86 | 82 | 77 | 77 | 78 | 85 |
| SBP (mmHg) | 97 | 94 | 84 | 97 | 85 | 98 | 97 | 93 | 96 | 98 | 90 | 94 | 100 | 97 | 102 | 82 | 89 | 87 | 84 | 80 | 83 | 79 | 74 | 82 | 81 | 92 | 90 | 89 | 85 | 83 | 84 | 95 | 94 | 94 | 98 |
| DBP (mmHg) | 58 | 57 | 54 | 58 | 58 | 56 | 63 | 59 | 55 | 60 | 55 | 60 | 61 | 60 | 64 | 50 | 51 | 57 | 59 | 56 | 54 | 55 | 53 | 51 | 56 | 56 | 60 | 54 | 54 | 56 | 66 | 60 | 63 | 63 | 69 |
| MBP (mmHg) | 71 | 69 | 64 | 71 | 67 | 70 | 74 | 70 | 68 | 72 | 66 | 71 | 74 | 72 | 74 | 60 | 63 | 67 | 67 | 64 | 63 | 63 | 60 | 61 | 64 | 68 | 72 | 65 | 64 | 65 | 78 | 71 | 73 | 73 | 72 |
| SpO2 (%) | 96 | 96 | 96 | 96 | 96 | 98 | 97 | 97 | 95 | 96 | 97 | 97 | 96 | 96 | 95 | 96 | 96 | 96 | 96 | 98 | 95 | 95 | 93 | 94 | 93 | 93 | 94 | 96 | 97 | 96 | 96 | 94 | 91 | 92 | 91 |
| RR (bpm) | 21 | 22 | 23 | 19 | 20 | 18 | 20 | 17 | 24 | 22 | 20 | 20 | 18 | 21 | 20 | 22 | 19 | 26 | 25 | 23 | 24 | 27 | 25 | 21 | 30 | 26 | 20 | 28 | 21 | 25 | 22 | 18 | 25 | 24 | 22 |
| HR, heart rate; SBP, systolic blood pressure; DBP, diastolic blood pressure; MBP, mean blood pressure; SpO_2_, peripheral capillary oxygen saturation; RR, respiratory rate | | | | | | | | | | | | | | | | | | | | | | | | | | | | | | | | | | | |
