# Supplementary material for: Acute myocardial infarction in a patient with HAM/TSP evaluated for circulatory dynamics during mobilization before and after antihypertensive drug modification: a case report
Source: J Med Case Rep. 2026 May 29;20:435. doi: 10.1186/s13256-026-06045-4 (PMC13422343; doi:10.1186/s13256-026-06045-4)
Supplement: Supplementary file 3 — Additional file 3: CARE checklist [file 13256_2026_6045_MOESM3_ESM.pdf]

CARE case report guidelines

CARE Checklist of information to include when writing a case report

(cc) BY-NC-ND

Topic	Item	Checklist item description	Reported on Line
Title	1	The diagnosis or intervention of primary focus followed by the words "case report"	1 – 3
Key Words	2	2 to 5 key words that identify diagnoses or interventions in this case report, including "case report"	66 – 67
Abstract (no references)	3a	Introduction: What is unique about this case and what does it add to the scientific literature?	37 - 43
	3b	Main symptoms and/or important clinical findings	46 – 49
	3c	The main diagnoses, therapeutic interventions, and outcomes	47 - 58
	3d	Conclusion—What is the main "take-away" lesson(s) from this case?	59 - 64
Introduction	4	One or two paragraphs summarizing why this case is unique (**may include references**)	70 - 103
Patient Information	5a	De-identified patient specific information.	106 – 113
	5b	Primary concerns and symptoms of the patient.	108 - 109
	5c	Medical, family, and psycho-social history including relevant genetic information	108 – 111
	5d	Relevant past interventions with outcomes	115 - 118
Clinical Findings	6	Describe significant physical examination (PE) and important clinical findings.	115 - 125
Timeline	7	Historical and current information from this episode of care organized as a timeline	151 - 154, Fig 1
Diagnostic Assessment	8a	Diagnostic testing (such as PE, laboratory testing, imaging, surveys).	115 - 125
	8b	Diagnostic challenges (such as access to testing, financial, or cultural)	245 – 251
	8c	Diagnosis (including other diagnoses considered)	116 – 118
	8d	Prognosis (such as staging in oncology) where applicable	223 - 231, 245 - 251
Therapeutic Intervention	9a	Types of therapeutic intervention (such as pharmacologic, surgical, preventive, self-care)	120 – 188
	9b	Administration of therapeutic intervention (such as dosage, strength, duration)	127 – 133
	9c	Changes in therapeutic intervention (with rationale)	134 – 142
Follow-up and Outcomes	10a	Clinician and patient-assessed outcomes (if available)	148 - 154, 184 - 188
	10b	Important follow-up diagnostic and other test results	162 - 175, 177 - 183
	10c	Intervention adherence and tolerability (How was this assessed?)	177 - 185
	10d	Adverse and unanticipated events	no adverse event
Discussion	11a	A scientific discussion of the strengths AND limitations associated with this case report	265 – 269
	11b	Discussion of the relevant medical literature **with references**.	197 - 264
	11c	The scientific rationale for any conclusions (including assessment of possible causes)	245 - 274
	11d	The primary "take-away" lessons of this case report (without references) in a one paragraph conclusion	271 – 274
Patient Perspective	12	The patient should share their perspective in one to two paragraphs on the treatment(s) they received	185 – 186
Informed Consent	13	Did the patient give informed consent? Please provide if requested	Yes ☑ No ☐
